# An Exonuclease I-Aided Turn-Off Fluorescent Strategy for Alkaline Phosphatase Assay Based on Terminal Protection and Copper Nanoparticles

**DOI:** 10.3390/bios11050139

**Published:** 2021-04-29

**Authors:** Yan Wang, Ying Yan, Xinfa Liu, Changbei Ma

**Affiliations:** School of Life Sciences, Central South University, Changsha 410013, China; 2801170213@csu.edu.cn (Y.W.); Yany2018@csu.edu.cn (Y.Y.)

**Keywords:** alkaline phosphatase, terminal protection, copper nanoparticle, fluorescence

## Abstract

As an important DNA 3′-phosphatase, alkaline phosphatase can repair damaged DNA caused by replication and recombination. It is essential to measure the level of alkaline phosphatase to indicate some potential diseases, such as cancer, related to alkaline phosphatase. Here, we designed a simple and fast method to detect alkaline phosphatase quantitively. When alkaline phosphatase is present, the resulting poly T-DNA with a 3′-hydroxyl end was cleaved by exonuclease I, prohibiting the formation of fluorescent copper nanoparticles. However, the fluorescent copper nanoparticles can be monitored with the absence of alkaline phosphatase. Hence, we can detect alkaline phosphatase with this turn-off strategy. The proposed method is able to quantify the concentration of alkaline phosphatase with the LOD of 0.0098 U/L. Furthermore, we utilized this method to measure the effects of inhibitor Na_3_VO_4_ on alkaline phosphatase. In addition, it was successfully applied to quantify the level of alkaline phosphatase in human serum. The proposed strategy is sensitive, selective, cost effective, and timesaving, having a great potential to detect alkaline phosphatase quantitatively in clinical diagnosis.

## 1. Introduction

Alkaline phosphatase (ALP), a ubiquitous enzyme found in human tissues such as the liver, intestine, bone, kidney, and placenta, is a homodimeric enzyme with necessary cofactors, including one magnesium atom and two zinc atoms [1,2]. Studies have shown that ALP is able to catalyze alkaline hydrolysis of nucleic acids, proteins, and some small molecules, which are phosphate [1,3,4,5]. Owing to the indispensable role in many physiological processes such as cell cycle, growth, apoptosis, and signal transduction, ALP is closely connected to multiple human diseases, especially bone and hepatic diseases [5,6,7]. The concentration of ALP in healthy people’s serum is 46–190 U/L [8]. Hence, any abnormal level of ALP in the serum may be an essential indicator of some diseases related to ALP function, such as diabetes, breast cancer, prostatic cancer, bone diseases, such as osteosarcoma, and hepatic diseases, e.g., Wilson’s disease. So ALP levels in the serum may be an effective biomarker in medical diagnosis [9,10,11]. Furthermore, Prakash et al. have recently found that the level of ALP in saliva (readily accessible, safe, and noninvasive body fluid) may be able to serve as an early biomarker for diabetes mellitus and some potentially malignant tumors [12]. In addition, ALP is capable of generating signals for the analytes by conjugating to streptavidin or the second antibody in the biological analysis, such as enzyme-linked immunosorbent assay (ELISA), histochemical staining, and aptamer-based assay [13,14]. As a result, the detection limit of analytes is highly dependent on the detection limit of ALP in these biological analyses. Besides, due to its ability to monitor phosphorus-related water eutrophication, ALP is a good indicator of algal boom [15]. Therefore, it is of great importance to developing a facile and sensitive method to detect ALP.

A number of diverse methods and techniques, including colorimetric assay, electrochemistry, chromatography, photometric assay, photoelectrochemical assay and surface-enhanced Raman scattering methods, have been developed to date to detect the concentration of ALP that catalyzes the dephosphorylation process of different substrates [16,17,18,19,20,21,22,23]. However, these traditional methods inevitably suffer from one or more limitations, such as time-consuming procedures, poor sensitivity, exorbitant material requirement, and use of complex devices. Recently, fluorometric methods have caught people’s eyes for their advantages such as simplicity, convenience, rapid response, and high sensitivity [24,25].

Due to the simplicity, high sensitivity, low cost, and rapidness, nanomaterial-based probes have attracted considerable attention lately [26]. On account of the fluorescence of some nanoparticles, including copper nanoparticles (CuNPs), silver nanoparticles (AgNPs), and gold nanoparticles (AuNPs), the signals can be monitored by spectrometers [27,28]. In recent years, some efforts have been made towards the detection of ALP by measuring the fluorescence intensity of nanoparticles. Chen and his coworkers successfully screened the concentration of ALP with a LOD of 0.125 U/L by constructing a sequentially triggered nanoswitch depending on CuNPs using single-stranded poly-(thymine) (poly T) DNA as a template [29]. Chen et al. reported that AgNPs formed by Ag+ and CdTe quantum dots could detect ALP with LOD of 0.25 U/L [30]. Lin et al. proposed a rapid method to detect ALP, based on redox-modulated silver deposition on AuNPs, with LOD of 0.52 U/L [31]. Among these assays, poly T DNA-templated CuNPs is remarkable owing to their simple procedure, less necessity of DNA templates, and rapid formation with the support of ascorbate to reduce Cu^2+^ within just a few minutes [32]. Moreover, poly T DNA-templated CuNPs are a prominent fluorescence probe, which exhibits a maximum λem at 615 nm with large MegaStrokes shifting with the ability to remove strong background signal from complex biological systems [33]. Considering that poly T DNA, an ideal template of fluorescent CuNPs, can be modified by the removal of a phosphate group from the 3′-end catalyzed by ALP, we have proposed a method for the measurement of ALP concentration with LOD of 0.0098 U/L requiring only 50 min based on terminal protection and fluorescent CuNPs. As far as we know, this is the first exonuclease I-aided turn-off fluorescent strategy for ALP assay based on terminal protection and CuNPs with high sensitivity in a short time.

## 2. Materials and Methods

### 2.1. Materials and Reagents

The alkaline phosphatase and exonuclease I were purchased from Takara Biotechnology Co., Ltd. (Dalian, China). Tris (hydroxymethyl) methyl aminomethane hydrochloric acid (Tris-HCl), magnesium chloride (MgCl_2_), sodium chloride (NaCl), 3′-(Nmorpholino) propanesulfonic acid (MOPS), copper sulfate (CuSO_4_), and sodium ascorbate were obtained from Sinopharm Chemical Reagent Co., Ltd. (Shanghai, China). Na_3_VO_4_ was bought from Shanghai Macklin Biochemical Co., Ltd. (Shanghai, China). The DNA probe is T30: 5′-TTTTTTTTTTTTTTTTTTTTTTTTTTTTTT-p-3′, it was synthesized by Shanghai Sangon Biotech Co. Ltd. (Shanghai, China). All the other reagents were of analytical grade.

### 2.2. Apparatus

The fluorescence emission spectra were recorded on Hitachi F-2700 fluorescence spectrophotometer (Hitachi Ltd., Tokyo, Japan) at the excitation wavelength of 340 nm that was obtained from 550 nm to 650 nm at room temperature. The resulting error was obtained from three repeated measurements, and statistical methods were used to collate and analyze the data during the experiment.

### 2.3. The Quantitative Detection of ALP

To measure the activity of ALP quantitatively, T30 (2 µM) and ALP solutions of different concentrations were added into Tris-HCl buffer, and the resultant solution was incubated at 37 °C for 10 min and 80 °C for 20 min. After that, Exo I (40 U/mL) was added to the above solution, and the resulting solution was incubated at 37 °C for 10 min. Subsequently, 73 µL of MOPS buffer, 0.36 µL of CuSO_4_ (28 mM), 1 µL of sodium ascorbate (500 mM) were added into the solution to get a final volume of 100 µL, and the resulting solution was incubated at room temperature for 10 min to form CuNPs. Finally, the fluorescence spectra of all the samples were recorded by the F-2700 fluorescence spectrophotometer.

### 2.4. Gel Electrophoresis Analysis

The 20% denaturing urea polyacrylamide gel electrophoresis (Urea-PAGE) in 1xTBE (89 mM Tris-boric acid, 2 mM EDTA, pH 8.3) was used to analyze the feasibility of the proposed method at 150 V for 105 min. Afterwards, silver staining was employed as the staining method to show the different products formed under different conditions.

## 3. Results

### 3.1. Sensing Strategy of ALP Detection

**Scheme 1:**
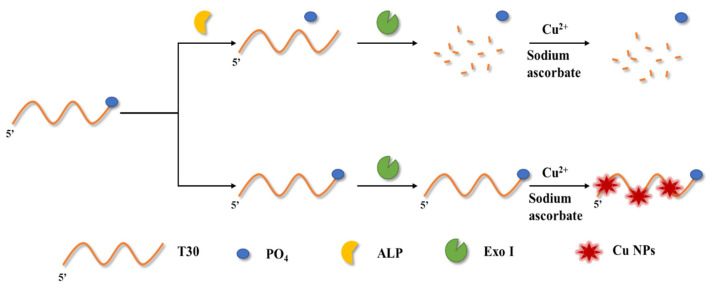
Schematic illustration of fluorescent ALP activity analysis based on DNA-templated CuNPs.

The method we proposed to measure the activity of ALP quantitatively is schematically illustrated in Scheme 1. A poly T-DNA with a phosphate modification at 3′-end is designated as the substrate of ALP. In the presence of ALP, the phosphate group is removed to liberate the phosphate group from the 3′-end so that there is a free 3′-OH which can be discerned by 3′ single-stranded-specific exonuclease I (Exo I) [34]. Afterwards, the poly T-DNA can be split off into small fragments losing its ability to act as the template of CuNPs despite the existence of Cu^2+^ and sodium ascorbate. On the contrary, in the absence of ALP, the poly T-DNA with a 3′-phosphoryl can’t be recognized by Exo I, so that integrated poly T-DNA becomes an ideal template for the formation of fluorescent CuNPs in the presence of Cu^2+^ and sodium ascorbate in the solution. Ultimately, the activity of ALP can be quantified by screening the fluorescent intensity changes.

### 3.2. Verification of the Feasibility of the Sensing Strategy

**Figure 1:**
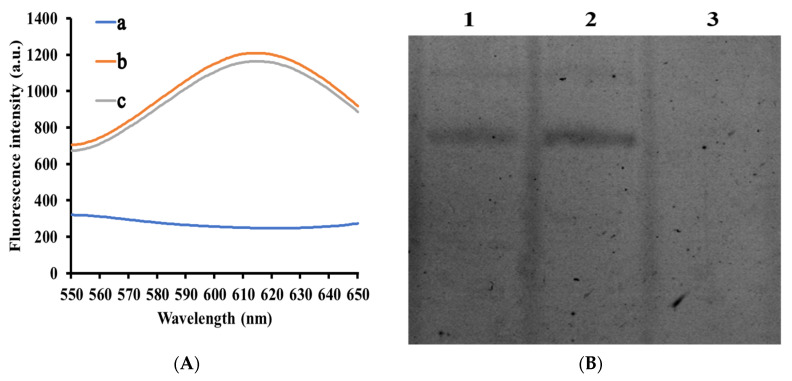
Investigation of the feasibility of the sensing strategy. (**A**) The fluorescence emission spectra of CuNPs under different conditions. Curve a: T30 + ALP + Exo I + Cu^2+^ + sodium ascorbate (T30, 2 µM; ALP, 500 U/L; Exo I, 40 U/mL; Cu^2+^, 200 µM; sodium ascorbate, 5 mM); curve b: without Exo I; curve c: without ALP. (**B**) Urea-PAGE with silver staining analysis under different conditions. Lane 1: T30+Exo I (T30, 2 µM; Exo I, 40 U/mL); lane 2: T30 (T30, 2 µM); lane 3: T30+ALP+Exo I (T30, 2 µM; ALP, 500 U/L; Exo I, 40 U/mL).

In order to demonstrate the feasibility of the proposed strategy, a series of experiments were carried out with or without ALP. As illustrated in Figure 1A, the CuNPs couldn’t form in the presence of ALP and Exo I (curve a). However, in the absence of ALP or Exo I, high fluorescence signals were observed (curve b, curve c). In addition, 20% Urea-PAGE stained by silver was employed to verify the feasibility of the proposed approach as well. Figure 1B shows that T30 disappeared in the presence of ALP and Exo I (lane 3). Nevertheless, when there was no ALP and Exo I (lane 2) or no ALP present (lane1), T30 could be stained by silver. These results verified the feasibility of the proposed strategy to detect ALP.

### 3.3. Optimization of Experimental Conditions

**Figure 2:**
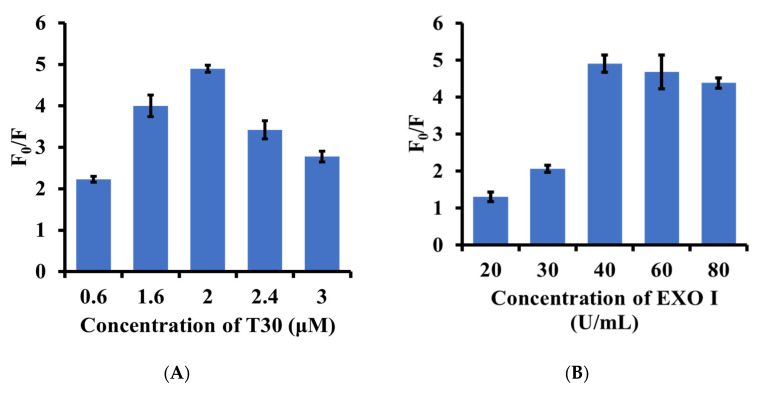

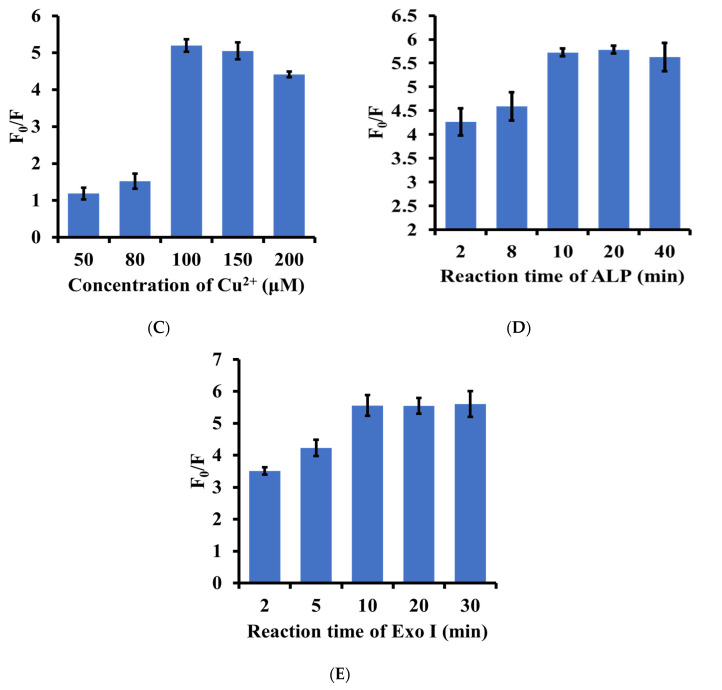
Optimization of reaction conditions. (**A**) The concentration of T30; (**B**) Exo I concentration; (**C**) Cu^2+^ concentration; (**D**) the reaction time of ALP; (**E**) the reaction time of Exo I.

We investigated the effects of different assay conditions, including the concentration of T30, the concentration of Exo I, the concentration of Cu^2+^, the reaction time of ALP, and the reaction time of Exo I. The fluorescence intensity ratios of the controlled group to the experimental group (F_0_/F) changed with varying assay conditions. As illustrated in Figure 2A–E, we found the optimal reaction condition as follows: 2 µM T30, 40 U/mL Exo I, 10 µM Cu^2+^ solution, a reaction time of 10 min between ALP and T30, and Exo I reaction time of 10 min.

### 3.4. Quantitative Fluorescence Measurement of ALP Activity

**Figure 3:**
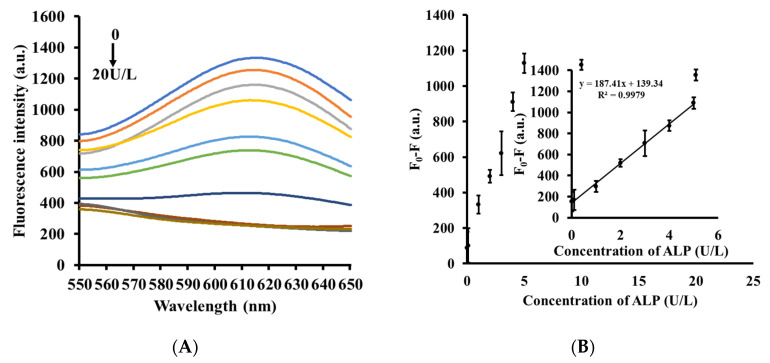
The quantitative measurement of ALP. (**A**) Fluorescence spectra of the assay system response to different activity units of ALP (0, 0.01, 0.1, 1, 2, 3, 4, 5, 10, and 20 U/L); (**B**) the plot between F_0_-F and the concentration of ALP. Inset: calibration linear curve for ALP detection.

In the present study, we have investigated the fluorescence responses of the proposed analytical method in varying concentrations of ALP under optimized conditions. As displayed in Figure 3A, the peak of fluorescence intensities decreased gradually when the concentrations of ALP increased from 0 to 20 U/L. The relationship between the F_0_-F [the fluorescence intensities (at 615 nm) of the controlled group minus the fluorescence intensities (at 615 nm) of the experimental group] and the activity of ALP is plotted in Figure 3B, where we can observe that F_0_-F values linearly increased with the concentration of ALP ranging from 0.01 to 5 U/L (regression coefficient R^2^ = 0.9979). The evaluated detection limit of the proposed strategy is 0.0098 U/L according to the 3σ rule, which is comparable or better than the existing methods (Table 1). Therefore, these results demonstrated the satisfactory sensitivity of the proposed method towards ALP.

### 3.5. Selectivity Assay

**Figure 4:**
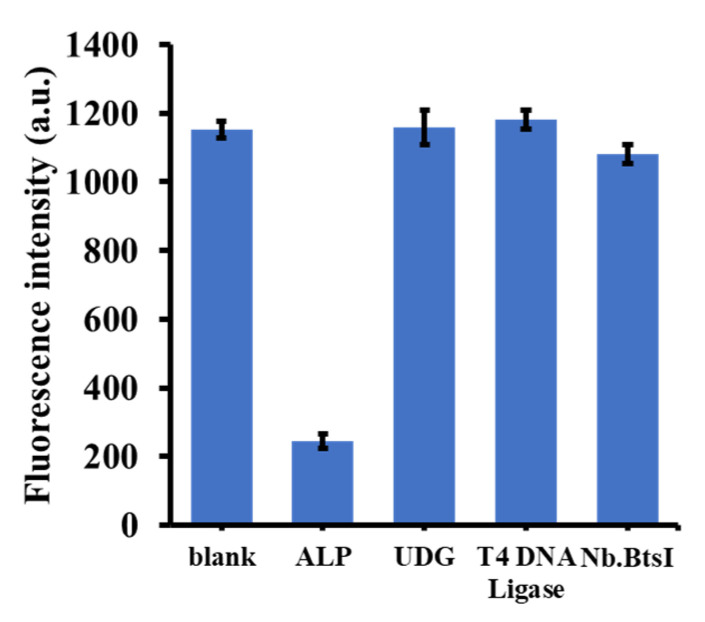
Selectivity assay. The concentrations of ALP, UDG, T4 DNA Ligase, and Nb. BtsI were 5 U/L.

To demonstrate the selectivity of the proposed strategy, the interfering proteins such as UDG, T4 DNA Ligase and Nb. BtsI, in the same concentration as ALP [13,14,36], were evaluated using the present method. As illustrated in Figure 4, the interfering proteins all arouse strong fluorescence responses except ALP, indicating the prominent specificity of the proposed approach.

### 3.6. ALP Inhibition Investigation

**Figure 5:**
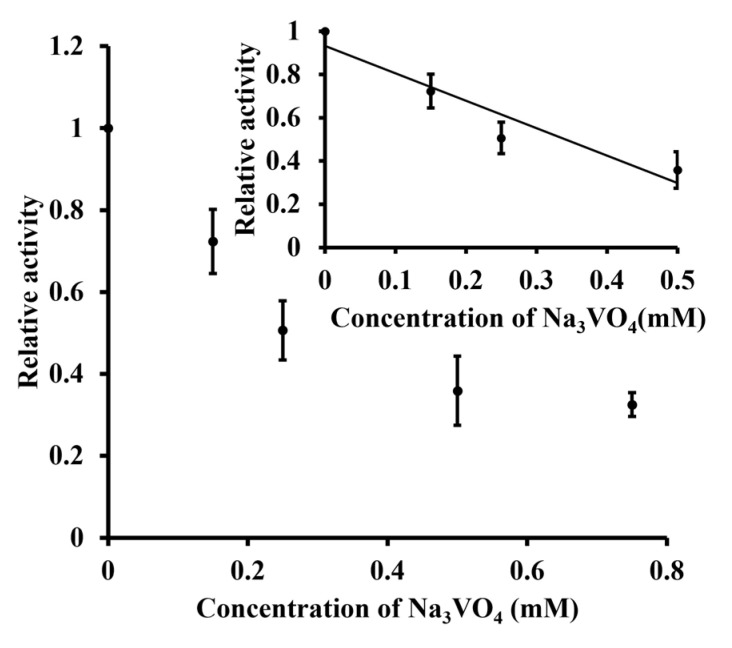
The inhibitory effects of Na_3_VO_4_ (0, 0.15, 0.25, 0.5, and 0.75 mM) on ALP. Concentration of ALP was 5 U/L. Inset: calibration linear curve for the inhibitory effects of Na_3_VO_4_ on ALP.

Na_3_VO_4_, reported to be one of the inhibitors of ALP, was chosen as the inhibitor to be applied. As shown in Figure 5, the value of relative activity of ALP decreased upon increasing the concentration of Na_3_VO_4_ from 0 to 0.75 mM. The inset graph indicated a linear relationship (R^2^ = 0.9126) between the relative activity and low Na_3_VO_4_ concentrations. The half-maximal inhibitory concentration (IC50) of Na_3_VO_4_ was calculated to be 0.433 mM. The results demonstrate that the proposed method can be applied to screen ALP inhibitors potentially.

### 3.7. ALP Assay in Diluted Human Serum Samples

To investigate the practical feasibility of the proposed strategy, a variety of concentrations of ALP, including 0.5 U/L, 3 U/L, and 5 U/L, were tested by the proposed method while adding a human serum into the reaction buffer to simulate the complex biological environment during the experimental procedures. As illustrated in Table 2, the recovery rates of various concentrations of ALP in 1% human serum diluted were 97.15% for 0.5 U/L, 102.11% for 3 U/L and 99.89% for 5 U/L with R.S.D of 7.95%, 8.73%, and 1.09%, respectively. Therefore, the results displayed that the proposed strategy has great potential in practical applications.

## 4. Conclusions

The proposed turn-off strategy shows high sensitivity, high selectivity with facile procedures in a short time in the quantification of ALP. Therefore, it has great potential to be utilized in the biological studies, early diagnosis and prognosis of some diseases related to the activity of ALP, such as diabetes, breast cancer, and prostatic cancer clinically [9,10,11]. However, there are still some challenges to overcome in practical applications. For example, this method requires a different reaction buffer which is a challenge in practical applications.

In conclusion, based on the poly T-DNA-templated formation of fluorescent CuNPs, we have proposed a facile but sensitive, selective, low-cost, and time-saving ALP assay. Besides, the assay depends on Exo I, which can only split off the one-stranded DNA with the 3′-hydroxyl end hydrolyzed by ALP. The proposed strategy showed superiority in a short time (40 min). The LOD value of 0.0098 U/L for the proposed assay demonstrates its high sensitivity. Compared with interfering enzymes, including UDG, T4 DNA Ligase, and Nb. BtsI without obvious variation of fluorescent signals, this method showed high selectivity to ALP. Moreover, when applied to test ALP levels in diluted human serum samples, with high recovery rates and low R.S.D, the proposed strategy showed its potentially practical value with satisfactory results. Given the simplicity, wonderful sensitivity, and high selectivity of the proposed method, we can envisage that it may find a wide application in clinical diagnosis and prognosis.

## Figures and Tables

**Table 1:** Comparison of different methods for the detection of ALP.

Methods	LOD (U/L)	Dynamic Range (U/L)	Reference
Colorimetric	0.52	3–18	[25]
Electrochemiluminescence	0.80	5–50	[20]
Photometric	0.68	1–210	[21]
Ratiometric assay	0.0017	0.005–60	[22]
Photoelectrochemical	0.06	0.1–4000	[23]
Electrochemical	0.20	3–50	[35]
Fluorescence AgNPs	0.25	1–1000	[30]
Fluorescence carbon dots	0.02	0.05–40	[4]
Fluorescence CuNPs	0.0098	0.01–5	This work

**Table 2:** Recovery experiments of ALP in human serum samples.

Sample	Added (U/L)	Found (U/L)	Recovery
1	0.5	0.49 ± 0.04	97.15%
2	3	3.06 ± 0.27	102.11%
3	5	4.99 ± 0.05	99.89%

## Data Availability

The data presented in this study are available on request from the corresponding authors.
